# Alteration of gene expression profiles during mycoplasma-induced malignant cell transformation

**DOI:** 10.1186/1471-2407-6-116

**Published:** 2006-05-04

**Authors:** Shimin Zhang, Shien Tsai, Shyh-Ching Lo

**Affiliations:** 1 Department of Environmental & Infectious Disease Sciences, Armed Forces Institute of Pathology, AFIP Building 54, Room 3025, 14^th ^Street and Alaska Avenue, NW, Washington, DC 20306-6000, USA

## Abstract

**Background:**

Mycoplasmas are the smallest microorganisms capable of self-replication. Our previous studies show that some mycoplasmas are able to induce malignant transformation of host mammalian cells. This malignant transformation is a multistage process with the early infection, reversible and irreversible stages, and similar to human tumor development in nature. The purpose of this study is to explore mechanisms for this malignant transformation.

**Methods:**

To better understand mechanisms for this unique process, we examined gene expression profiles of C3H cells at different stages of the mycoplasma-induced transformation using cDNA microarray technology. A total of 1185 genes involved in oncogenesis, apoptosis, cell growth, cell-cycle regulation, DNA repair, etc. were examined. Differences in the expression of these genes were compared and analyzed using the computer software AtlasImage.

**Results:**

Among 1185 genes screened, 135 had aberrant expression at the early infection stage, 252 at the reversible stage and 184 at the irreversible stage. At the early infection stage, genes with increased expression (92 genes) were twice more than those with decreased expression (42 genes). The global gene expression at the reversible stage appeared to be more volatile than that at any other stages but still resembled the profile at the early infection stage. The expression profile at the irreversible stage shows a unique pattern of a wide range of expression levels and an increased number of expressing genes, especially the cancer-related genes. Oncogenes and tumor suppressors are a group of molecules that showed significant changes in expression during the transformation. The majority of these changes occurred in the reversible and irreversible stages. A prolonged infection by mycoplasmas lead to the expression of more cancer related genes at the irreversible stage.

**Conclusion:**

The results indicate that the expression profiles correspond with the phenotypic features of the cells in the mycoplasma induced transformation process. The early mycoplasma infection stage shares a common phenomenon with many other acute infections, genes with increased expression significantly outnumbering those with decreased expression. The reversible stage is a transition stage between benignancy and malignancy at the molecular level. Aberrant expression of oncogenes and tumor repressors plays a key role in mycoplasma-induced malignant transformation.

## Background

Mycoplasmas are a group of the smallest free-living organisms that commonly infect or colonize the oral-respiratory and urogenital tracts of humans and animals. These wall-free mycoplasmas are among the few prokaryotes that can grow in close interaction with mammalian cells, often silently, for a long period of time [1]. Our previous studies have revealed that chronic and persistent infection with these seemingly low-virulence mycoplasmas could gradually but significantly affect many important biological characteristics of mammalian cells and even lead to malignant transformation [2-4].

C3H cells (C3H10T1/2), a murine embryonic cell line, rarely undergo spontaneous transformation, and are one of the few standard test systems for studying potential carcinogenic agents in animals or humans [5]. *M. fermentans *and *M. penetrans *are the most common mycoplasmas associated with HIV infection or AIDS, and have been proposed to play a co-factor role in the disease process [6]. Our earlier study revealed that chronic infections with *M. fermentans *and *M. penetrans *induced malignant transformation of C3H cells [3]. However, as opposed to earlier reports of mycoplasma-associated malignant cell transformation [7,8], our study showed that infection by *M. fermentans *or *M. penetrans *did not lead to rapid transformation of C3H cells. Instead, the mycoplasma-mediated malignant cell transformation required a prolonged period of infection with these organisms (a long latency) and was evidently a multistage process. Although C3H cells showed prominent morphological transformation after 7 weeks of mycoplasmal infections, malignant properties associated with cell transformation could be rapidly reversed by eradicating the mycoplasma from culture with antibiotics up to 11 weeks of mycoplasmal infection (reversible stage). However, further persistent infection beyond 18 weeks resulted in an irreversible form of transformation that included prominent chromosomal alteration, the ability to form tumors in animals and high efficiency of colony formation in soft agar. At this irreversible stage, the transformed cells showed unique karyotypic changes and growth characteristics. The continued presence of the transforming mycoplasmas was no longer required for maintaining the transforming traits of the cells [3].

The multistage transformation effect associated with chronic *M. fermentans *and *M. penetrans *infections was similarly observed in our recent studies using 32D cells, a mouse hematopoietic cell line strictly dependent on IL-3 for their growth [2,9]. Infection with *M. fermentans *or *M. penetrans *prevented apoptosis and effectively induced IL-3 independent growth of the cells. Prolonged infections with these mycoplasmas eventually lead to autonomous growth of the cells *in vitro *and to form tumors in mice in the absence of either IL-3 or the transforming mycoplasmas [2,10]. Some strains of *M. fermentans *were very effective in immortalization of human peripheral blood mononuclear cells [11], which might be linked with some hematological malignancies.

The long latency and distinct multistage process of malignant cell transformation induced by the human mycoplasmas closely resemble human tumor development in nature. It has provided an excellent model system for studying molecular mechanisms of malignant cell transformation. Our earlier studies of possible molecular mechanisms revealed that over-expression of H-ras and c-myc oncogenes was associated with malignant transformation of C3H cells [12]. On the other hand, no evidence of integration of mycoplasmal genetic material (DNA) could be found in the malignant C3H cells induced by mycoplasmal infections [13]. Since many different genes are likely involved in the malignant cell transformation, a global analysis of the gene expression at the different stages of mycoplasma-induced malignant transformation is highly desired. The recently developed cDNA microarray technology would allow us to effectively analyze the expression of many genes in a short period of time [14,15]. This technology has been used to analyze gene expression in host cells infected with various microorganisms [16,17], in virus transformed mammalian cells [18] and in cancer tissues [19]. In this study, we used the cDNA microarray technique to investigate the global changes of the gene expression in C3H cells at the different stages of the mycoplasma-induced malignant transformation. The array analyzed changes in expression of 1,185 different genes. Many of them have been proven to play an important role in cell malignant transformation.

## Methods

### 1. Induction of malignant transformation of C3H cells

Malignant transformation of mouse embryonic C3H cells by persistent infection of *M. fermentans *incognitus stain (MI) was previously described in detail [3]. In this study, we used parallel cultures of noninfected C3H cells as the control, C3H cells infected with *M. fermentans *for 1 week, 11 and 18 weeks as representatives of the early mycoplasma infection stage and the reversible as well as irreversible stages of the mycoplasma-induced malignant transformation, respectively. RNAs prepared from these cells were used in the following analysis of gene expression profiles.

### 2. RNA preparation and quantification

Total RNA was prepared from C3H cells using TRIzol reagent (Life Technologies, Inc., Gaithersburg, MD) according to manufacturer's instructions [20]. Total RNA prepared from mycoplasma-infected and non-infected control C3H cells were treated with RNase-free DNase I (Roche Diagnostics Corporation, Indianapolis, IN) to remove residue genomic DNA. The RNA was then extracted with phenol/chloroform/isoamyl alcohol followed by ethanol precipitation. The integrity and concentration of the purified RNA were further determined by measuring the absorbance at 260 nm and the 260/280 absorbance ratio on a spectrophotometer and by electrophoresis on a 1.2% agarose gel in formaldehyde-MOPS (3- [N-Morpholino]propanesulfonic acid) buffer [4].

### 3. Detection of gene expression using the cDNA microarray technique

To study global gene expression in C3H cells during the malignant transformation process, radioactively labeled cDNA prepared from DNase treated total RNA of C3H cells was selectively hybridized onto high density arrays of membrane-bound double strand cDNAs on the Atlas Mouse Cancer 1.2 Array (Clontech Laboratories, Inc., Palo Alta, CA). This array contains cDNA of 1,185 different genes known to be actively involved in cell transformation, cell adhesion and mobility, cell cycle, stress response, DNA damage repair, transcription, translation, signal transduction, basic metabolism, trafficking/transportation/targeting as well as extracellular matrix proteins. The detail description of the array information could be found in Clontech's Atlas Mouse Cancer 1.2 Array user manual.

Preparation of radioactively labeled cDNA probes and subsequent hybridization to the cDNA arrays were conducted as recommended by the manufacturer, using reagents provided by the manufacturer. In brief, for each array, 2.5 μg of total RNA were reverse-transcribed with MMLV reverse transcriptase in the presence of 35 μCi of [α-P^32^] dATP. The radioactive cDNA probes were purified using NucleoSpin extraction spin columns. The probes with total cpm >2 × 10^6 ^were used for the subsequent hybridization. The hybridization of the radioactively labeled probes to the Atlas arrays was conducted in rolling tubes in a Micro Hybridization Oven at 68°C overnight. The arrays were washed with 2× SSC containing 1% SDS 4 times and with 0.1 × SSC containing 0.5%SDS once at 68°C, each for 30 minutes, followed by a final wash with 2× SSC at room temperature for 5 min. The membranes were exposed to BioMax X-ray films with BioMax Transcreen HE intensifying screens (Eastman Kodak Company, Rochester, NY). The films were then developed and scanned on an Epson scanner.

The hybridization of the probes prepared from total RNA of C3H cells at different transformation stages to cDNA microarrays of the same batch was carried out under identical conditions. Subsequent wash steps, generation of image data and data analysis were performed in parallel. This approach facilitated the direct comparison of gene expression level between mycoplasma-infected cells and noninfected control cells.

### 4. Array data analysis

The Image data from microarray hybridization were further analyzed using AtlasImage™ 2.01 software (Clontech Laboratories, Inc.). A gene with a specific intensity (signal intensity minus local background intensity) higher than 5000 density units (cutoff level) was defined as expressed. In order to compare the gene expression profile of two arrays, global normalization was calculated by the sum method. Differences of signal intensity minus background on each array were added for all values over background. Afterwards, the normalization coefficient was determined by calculating the ratio of array 1 sum and array 2 sum. After normalization, background was subtracted. The adjusted intensity of a corresponding gene on two arrays was compared. The ratio threshold was set at 2 and the difference threshold was set at 5000 units.

### 5. Materials and chemicals

Cell culture medium RPMI 1640 and fetal bovine serum were purchased from Life Technologies, Inc. SP4 medium for mycoplasma culture was prepared in our laboratory [21]. Tissue culture plastic wares were from Nalge Nunc International, Rochester, NY. [α-^32^P] dATP (specific activity >3,000 mCi/mM) was from ICN Biomedicals, Inc. Aurora, OH. Ciprofloxacin was from Bayer Corporation, West Haven, CT. All general chemicals were purchased from Sigma-Aldrich, St. Louis, MO.

## Results

### Gene expression in C3H cells at different stages of malignant transformation induced by mycoplasmal infections

An Atlas cDNA expression membrane microarray, Mouse Cancer 1.2 Array, was used to study expression profiles of genes known to be associated with oncogenesis in C3H cells at the different stages of mycoplasma-induced malignant transformation. Each gene was represented on the array as one spot containing immobilized cDNA fragments. ^32^P-cDNA probes synthesized by the *in vitro *reverse transcription from total RNA of C3H cells infected with mycoplasmas for different periods of time and from non-infected control cells were hybridized to the arrays. Analysis of signal intensity on the arrays showed that transcripts (mRNA) of 736 genes (62.1%) out of 1185 arrayed genes were scored as expressed in non-infected control cells (Tab. 1). The gene expression of control C3H cells has been measured 3 times using the same batch of RNA and cDNA microarrays. The same expression pattern was observed. The expression of 755 genes (63.7%) was detected in the cells infected with *M. fermentans *for 1 week (early infection stage), 748 genes (63.1%) in the cells infected for 11 weeks (reversible stage) and 862 genes (72.9%) in the cells infected for 18 weeks (irreversible stage). The specific intensity of the expressed gene spots on the array ranged from 5008 to 65280 arbitrary units above background. The irreversibly transformed C3H cells (C3H/MI/P18) expressed 15% more genes than non-infected control cells or cells at other stages of the mycoplasma-induced malignant transformation.

### Differential profiles of gene expression in control cells and cells at different stages of the malignant transformation

Comparison of gene expression profiles between non-infected control cells and mycoplasma-infected C3H cells at different stages of transformation revealed that 92 genes (7.76% of arrayed genes) were significantly up-regulated and 43 genes (3.63%) were down-regulated in C3H cells following one week of mycoplasmal infection. After 11 weeks of mycoplasmal infection, 138 genes (11.65%) became up-regulated and 114 genes (9.62%), down-regulated. Numbers of genes with increased expression and decreased expression were 113 (9.53%) and 69 (5.82%), respectively, in cells infected with the mycoplasmas for 18 weeks. The distribution of genes with altered expression at different stages of cell transformation is shown in Table 1, according to their associated functions.

**Figure 1:**
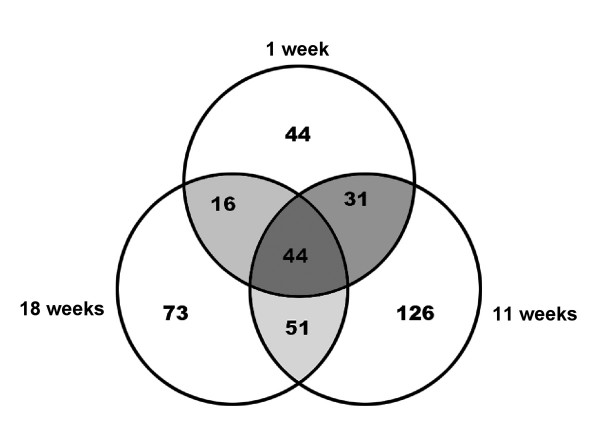
Venn diagram of genes with aberrant expression in C3H cells at different stages of the mycoplasma-induced malignant transformation. Circles symbolize individual stages of mycoplasma-induced malignant transformation. Numbers in a circle stand for genes expressed at that stage at least 2 fold differentially when compared to those expressed in control cells, while numbers in shared areas of circles represent genes with altered expression occurred at those stages.

**Figure 2:**
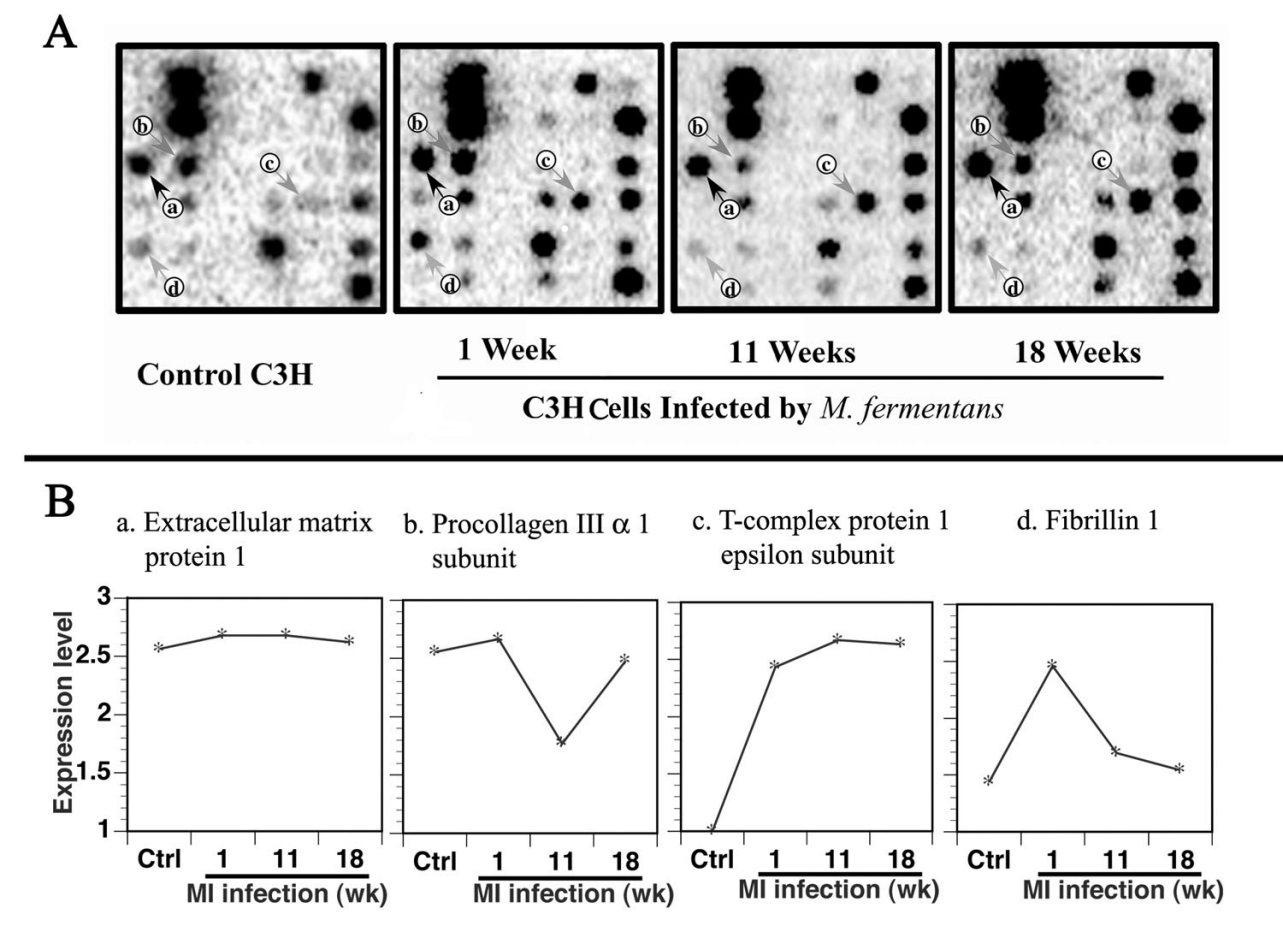
Kinetic changes in the gene expression during mycoplasma-induced transformation. Total RNAs were prepared from C3H cells infected with *M. fermentans *for 1 week (wk), 11 weeks (wks) and 18 wks as well as from noninfected control cells (Ctrl). Radioactive cDNA probes prepared from these RNAs were hybridized to the Mouse Cancer 1.2 Arrays. Results were obtained by the autoradiography. Four autoradiographic pictures in Panel A represent the same areas on the arrays hybridized with cDNA probes prepared from control cells and cells infected with the mycoplasmas for 1 wk, 11 wks and 18 wks. Dots pointed by arrows are individual genes: a. Extracellular matrix protein 1; b. Procollagen III α1 subunit; c. T-complex protein 1 epsilon subunit and d. Fibrillin 1. Panel B is graphic depiction of kinetic changes of the expression of the above 4 genes during mycoplasma-induced transformation process. Scale on Y axis is the relative gene expression level and X axis is different stages of the mycoplasma induced transformation.

Further analysis of the gene expression in C3H cells revealed that the expression of 44 genes was altered in cells at all three stages of malignant transformation following mycoplasmal infections. Some changes of the gene expression occurred in cells at two different stages and others only at a single stage of the transformation (Fig. 1). Compared with the gene expression in control cells, more genes are up-regulated in mycoplasma-infected cells. The majority (97.02%) of the alterations that occurred in two or three stages had the same direction (either increased or decreased expression). An example of differential expression of C3H cells during mycoplasma-induced malignant transformation is shown in Fig. 2. Expression of many genes, such as extracellular matrix protein 1, remained unchanged at the different stages of the mycoplasma-induced transformation (Fig. 2, a). The mRNA level of procollagen III α1 subunit decreased transiently in cells infected by mycoplasmas for 11 weeks (Fig. 2, b). The expression of fibrillin 1, on the contrary, markedly increased in cells infected by mycoplasmas for 1 week then returned to the original low level in the later phase of mycoplasmal infection (Fig. 2, d). On the other hand, the expression of T-complex protein 1 epsilon subunit was quickly elevated at the early stage of mycoplasmal infection and remained high in C3H cells infected by the mycoplasmas for 18 weeks (Fig. 2, c).

### Global comparison of gene expression profiles among control cells and cells at different stages of mycoplasma-induced transformation

**Figure 3:**
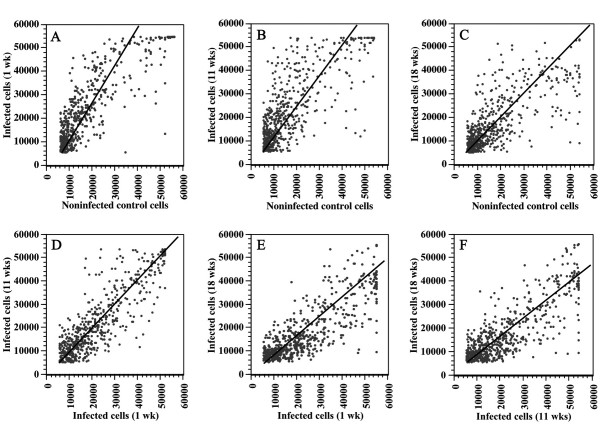
Comparison of gene expression profiles among control C3H cells and *M. fermentans *infected cells at different stages of the malignant transformation. Gene expression in the cells was screened by the cDNA microarray technique. Expression profiles were generated by the analysis of the gene expression data using the computer software AtlasImage™ version 2.01. A comparison of these profiles was made among the control cells and infected cells. The graphs were plotted as the expression level at one stage over that at another stage for each of the 1185 genes examined. Correlation lines were plotted by the computer-fitting. The scales on both X- and Y-axis represent gene expression level using arbitrary density units of each dot. Panel A, Control cells vs. cells infected for 1 week (wk); Panel B, Control cells vs. cells infected for 11 weeks (wks); Panel C, Control cells vs. cells infected for 18 wks; Panel D, Cells infected for 1 wk vs. for 11 wks; Panel E, Cells infected for 1 wk vs. for 18 wks and Panel F, Cells infected for 11 wks vs. for 18 wks.

In order to search for characteristic gene expression profiles of C3H cells for individual stages during the mycoplasma transformation, gene expression levels were globally compared among control cells and cells infected with the mycoplasmas for different lengths of time. As a result, the expression levels for each individual gene in C3H cells from 2 differently treated cultures formed a single dot (expression signature) at the cross point on scatter graphs. If expression levels of a given gene from two different samples are the same, the expression signature should be on the 45° hypothetic line of the chart. If the expression profiles from two different samples are similar, the correlation line that is plotted from all expression signatures should be near 45° and all signatures should be on or close to this line (Fig. 3).

The scattered dots of the gene expression from 2 different batches of control cells that were cultured for different periods of time were curve-fitted and showed a good linear correlation around 45° and a high correlation coefficient, which represent a high similarity of the gene expression profiles between the two batches of control cells. However, the gene expression profiles were significantly different between the non-infected control cells and the cells infected *by M. fermentans *for different periods of time (Fig. 3). At the early stage of mycoplasmal infection, the number of genes (92) with increased expression was twice more than that (43) with decreased expression (Table 1). This caused the left shift of the correlation line on the scatter plot (Fig. 3A). The correlation coefficient (r) was 0.695. However, the expression signatures between 1 week-infected and the non-infected control C3H cells did not scatter widely. The expression signatures on the scatter graphs between control cells and infected cells were increasingly more scattered and the correlation line gradually right-shifted with continuing mycoplasmal infection (Fig. 3B and 3C). The correlation coefficients between control cells and cells infected for 11 weeks and 18 weeks were 0.602 and 0.585 respectively. Surprisingly, a high correlation (r = 0.784) in the gene expression profile was observed between cells infected with mycoplasmas for 1 week and 11 weeks (Fig. 3D). The correlation line was near 45°. By contrast, more significant differences in the expression profiles between cells infected for 1 week and 18 weeks (Fig. 3E, r = 0.602) as well as between cells infected for 11 weeks and 18 weeks (Fig. 3F, r = 0.698) were observed.

### Alteration in the expression of cancer-associated genes during mycoplasma induced malignant transformation

We specifically examined the expression profile of various oncogenes and tumor suppressor genes. Among a total of 65 oncogenes and tumor suppressor genes screened, 43 were expressed in the non-infected control C3H cells. The number of expressed oncogenes and tumor suppressor genes was 46 in cells infected with the mycoplasmas for 1 week and 41 in cells infected with the mycoplasmas for 11 weeks. However, the number was significantly increased in the irreversibly transformed cells. Fifty-three oncogenes and tumor suppressors were expressed in the C3H cells following 18 weeks of mycoplasmal infection (Table 1). Compared to the expression level of these cancer-associated genes in control cells, one oncogene (myeloblastosis oncogene-like 2) was over-expressed and one (transformation-related protein 53, Trp 53 or p53) was under-expressed in cells at the early infection phase. Expression of two other oncogenes (G-protein coupled receptor 25 and chemokine-like receptor 1) was up-regulated from non-expression level in control cells to a level 3 fold higher than the cutoff expression level at the early infection stage and maintained at this high level of expression throughout the entire transformation process. Expression of the tumor suppressor p53, a most extensively studied tumor suppressor, was decreased in this entire transformation process induced by mycoplasmal infection. The number of oncogenes/tumor suppressor genes with detectable expression in cells infected with mycoplasmas for 11 weeks (41 genes) appeared to be fewer than those expressed at other stages of mycoplasmal infection. However, the number of genes with aberrant expression in this particular stage was the highest among the three stages. Six genes were up-regulated and 7 down-regulated in this stage (Table 2). In the 18-week mycoplasma-infected cells, 6 oncogenes and 2 tumor suppressor genes were over-expressed, 1 oncogene and 1 tumor suppressor gene were under-expressed (Table 2).

## Discussion

The present study attempts to further elucidate the molecular mechanisms that may play a critical role in the complex malignant cell transformation process, using a mycoplasma-oncogenesis model we previously developed. The *in vitro *model system shares many important characteristics found in the development of human cancer, including long latency and multistages of progression [3]. The newly available microarray technique allows us to compare the expression profiles of more than 1000 genes that are likely to be associated with oncogenesis in cells at different stages of malignant transformation induced by mycoplasmal infection. Two groups of molecules, the oncogenes and tumor suppressor genes, and apoptosis-associated genes, which could be of particular importance in the development and progression of malignant tumors, were included in this study.

At the early stage of mycoplasmal infection (the 1st week), C3H cells responded with a gene expression profile often seen in cells following acute microbial infections [22-25], many more genes with increased expression than genes with decreased expression. As a result, a significant left shift of the correlation line on the scatter graph of the gene expression was noted in Fig. 3. About half of the over-expressed genes were related to cell signaling (receptors, cell signaling and intracellular transducers/effectors/modulators, Table 1). Thus, despite the seemingly unapparent infections of mycoplasmas in culture (and *in vivo*), mammalian host cells evidently had a strong response and produced a host of signaling molecules to coordinate messages that were important for the cells' adaptation to the new environment.

C3H cells at the reversible stage of transformation, following 11 weeks of mycoplasmal infection, were apparently in a transitional phase between benignancy and malignancy. Eradicating the transforming mycoplasmas from the culture with antibiotics could reverse all the malignant phenotypes of C3H cells. The gene expression profile of C3H cells at this specific stage was more volatile and appeared to reflect a transitional phase with more genes having aberrant expression than at any other stages during the mycoplasma-induced malignant transformation. On the other hand, a comparison of gene expression levels between cells at the early mycoplasma infection stage and at the reversible stage revealed that the correlation line was at nearly 45° and expression signatures (dots) were distributed relatively close to the line (Fig. 3D). This scatter distribution of the gene expression signatures suggested that the gene expression pattern at the reversible stage in general still mostly resembled that at the early mycoplasma infection stage. Many studies have shown that malignant phenotypes induced by quantitative changes of oncogene(s) could be reversed by interrupting the activation of causative oncogene(s) [26]. Our earlier study revealed that eradication of mycoplasmas from cell cultures with an antibiotic would abolish the aberrant expression of c-myc and H-ras and reverse all of the malignant properties of C3H cells at this stage [12]. These data support our theory that malignant phenotypes of the cells at the reversible stage might be caused by quantitatively aberrant expression of certain oncogenes and tumor suppressors in the response to chronic mycoplasmal infection.

C3H cells became irreversibly or permanently transformed following 18 weeks of mycoplasmal infection and gained the ability to form tumors in animals and colonies in soft agar. These cells were no longer dependent on the continuing presence of mycoplasmas for maintaining their malignant properties [3]. Our present study revealed that cells at this stage had the highest gene expression rate among the 3 different stages in the transformation process (Table 1). However, the number of genes with altered expression actually decreased 27% from that at the reversible stage (Table 1). A comparison of gene expression profiles between control cells and cells at the 3 three progressive stages of the transformation showed that the correlation lines on the scattered gene expression graphs were gradually right-shifting (Fig. 3A, 3B and 3C). The correlation coefficients were gradually decreasing. Although the correlation line in the gene expression scatter plot between the control cells and cells at the irreversible stage was near 45° (Fig. 3C), the expression signatures were more scatter than those at any other stages of the transformation process. These collective results suggest that the gene expression profile at the irreversible stage might be changed from a volatile status at the reversible stage to a relatively stable status that is far more different from that of the noninfected control cells. The newly established gene expression pattern at the irreversible stage may provide essential material basis for a new set of interactions of molecules, which support the stable malignant phenotypes of the transformed cells.

The cells at the irreversibly transformed stage also had the highest expression rate of cancer-related genes during the mycoplasma-induced malignant transformation (Table 1). Increase of oncogene expression and decrease of tumor suppressor genes expression at the reversible stage (Table 2) seems to provide a relatively straightforward explanation for the mycoplasma mediated malignant transformation. However, we also found decreased expression of 4 oncogenes at the reversible stage and increased expression of one tumor suppressor at the irreversible stage. A similar phenomenon was also observed in the expression profile of apoptosis associated genes (Data not shown). Mutation(s) in these molecules could be responsible for the decreased oncogene expression and increased tumor suppressor gene expression in the transformed cells. The mutation could alter physiological functions of tumor- and apoptosis-associated genes. The host cells might compensate the normal functions of the affected molecules by adjusting their expression levels. This might be the case in the mycoplasma-induced transformation. Chronic mycoplasmal infection could cause the genetic instability and changes in the genetic material (DNA) of host cells [2,3,11].

There are tens of thousand of different molecules and hundreds of biochemical pathways in mammalian cells. Over-expression or down-regulation of a single molecule that plays an important role in the control of cell proliferation could evidently alter the balance of the entire cell physiological network and lead to malignant transformation [27,28]. We believe that prolonged over-expression and/or down-regulation of a group of important molecules would likely change the critical balance of physiological networks in cells chronically infected by mycoplasmas and result in malignant cell transformation. The present microarray study has allowed us to identify changes of expression (up-regulated and down-regulated) of many important oncogenesis-associated genes at the different stages of mycoplasma-mediated cell transformation. We believe that the pattern of gene expression profile alterations observed in our mycoplasma-induced transformation model might be similar to those found in many transformation models induced by transforming microorganisms. However, due to the differences between the transforming microorganisms, alterations of many individual genes observed in this study could be unique to the mycoplasma-induced transformation process. Therefore, more work is needed to identify genes specific for mycoplasma-induced transformation and which genes are truly responsible for the progression of this complex process. The further challenge is to elucidate how the aberrant expression of these genes in cells would cause permanent or constitutive activation of certain oncogenes and leads to the irreversible malignant transformation.

## Conclusion

This study reveals different gene expression profiles of C3H cells at different stages of the mycoplasma-induced malignant transformation. The results indicate that these expression profiles correspond with the phenotypic features of the cells in the mycoplasma induced transformation process. The early mycoplasma infection stage shares a common phenomenon in the gene expression profile with many other acute infections, genes with increased expression outnumbering those with decreased expression. The reversible stage is relatively unstable and chaotic with the highest number of altered genes. Its profile reflects the nature of the transition between benignancy and malignancy at the molecular level. The expression profile at the irreversible stage shows a unique pattern of a wide range of expression levels and an increased number of expressing genes, especially the cancer-related genes. Aberrant expression of oncogenes and tumor repressor might play a key role in the mycoplasma-induced malignant transformation.

## Competing interests

The author(s) declare that they have no competing interests.

## Authors' contributions

SZ; designed and performed the study, and prepared the manuscript. ST; performed experiments of mycoplasma-induced transformation. SL; was involved in designing the study and revising the manuscript.

## Pre-publication history

The pre-publication history for this paper can be accessed here:



## Figures and Tables

**Table 1:** Differential gene expression between control cells and cells at different stages of mycoplasma-induced malignant transformation

Gene Class	Number of genes										
	
	Screened	Expressed in control cells	Expressed and altered in cells following mycoplasmal infection								
			
			1 week			11 weeks			18 weeks		
			
			Expressed	Increased	Decreased	Expressed	Increased	Decreased	Expressed	Increased	Decreased
Apoptosis associated proteins	48	30	34	4	2	27	3	7	25	6	5
Cell adhesion receptors/proteins	100	61	60	6	1	61	9	10	69	12	3
Cell cycle associated proteins	45	34	31	2	4	26	2	9	35	2	5
Cell receptors (by activities)	162	74	90	16	5	90	26	16	70	9	14
Cell signaling, extracellular communication proteins	144	89	82	8	4	98	18	10	112	11	6
Cytoskeleton/motility proteins	59	39	41	4	3	37	8	8	51	6	7
DNA binding and chromatin proteins	44	23	29	5	1	29	6	1	31	8	0
Extracellular transport/carrier proteins	6	4	5	0	0	3	1	0	5	0	0
Extracellular matrix proteins	30	28	29	3	2	29	4	1	27	2	7
Functional unclassified	15	8	11	3	1	13	5	1	13	2	1
Intracellular transducers/effectors/modulators	253	155	158	20	6	149	26	28	201	32	10
Membrane channels, transporters and cell surface antigens	27	16	16	4	1	20	7	0	18	4	1
Metabolism-associated proteins	26	14	16	5	1	18	4	2	20	3	0
Oncogenes and tumor suppressors	65	43	46	3	1	41	6	7	53	8	2
Stress response proteins	39	24	22	1	1	21	3	2	26	2	3
Trafficking/targeting proteins	5	2	1	0	0	2	0	0	3	0	0
Transcription associated proteins	30	27	20	3	4	25	2	3	29	1	0
Translation, post-translation modification and protein turnover	87	65	64	5	6	58	8	9	74	7	5
Total	1185	736	755	92	43	748	138	114	862	115	69

**Table 2:** Alterations of the expression of oncogenes and tumor suppressors in C3H cells during mycoplasmal induced malignant transformation

Gene/Protein Name	Classification		Change of the expression at different stages		
			
		GenBank	1 wk	11 wks	18 wks
Casitas B-lineage lymphoma	Oncogene	X57111			2.23
Villin 2	Oncogene	X60671			2.15
FBJ osteosarcoma oncogene	Tumor suppressor	V00727			Down
G-protein coupled receptor 25	Oncogene	U39827	Up	Up	Up
Chemokine-like receptor 1	Oncogene	U79525	Up	Up	Up
SWI/SNF related, matrix associated, actin dependent regulator of chromatin, subfamily b, member 1	Tumor suppressor	AJ011739		0.49	
Notch gene homolog 4 (Drosophila)	Oncogene	M80456		Down	
Lymphoid blast crisis-like 1	Oncogene	U28495			Up
Myeloblastosis oncogene-like 1	Oncogene	L35261		Down	
Myeloblastosis oncogene-like 2	Oncogene	X70472	3.20	2.94	
Neurofibromatosis 2	Tumor suppressor	L27105			2.65
Retinoblastoma-like 1 (p107)	Tumor suppressor	U27177		Down	
Transformation related protein 53	Tumor suppressor	K01700	0.39	0.22	0.42
RAB17, member RAS oncogene family	Oncogene	X70804		Up	
RAB24, member RAS oncogene family	Oncogene	Z22819		Up	Up
Src homology 2 domain-containing transforming protein C1	Oncogene	U15784		Down	
SKI-like	Oncogene	U36203		Down	
Vav 1 oncogene	Oncogene	X64361		Up	Up

1. The comparison was made by dividing the gene expression level in control cells by that in cells infected with mycoplasmas for 1 week (wk), 11 weeks or 18 weeks (wks).

2. The ratio ≥ 2.0 or ≤ 0.5 was defined as a significant increase or decrease when expression levels of a given gene in both samples were greater than the cutoff level, 5000 density units.

3. "Up" means that the adjusted intensity of a gene in control cells is below 5000 units (a cutoff line for expression), while the adjusted intensity of this gene in mycoplasma infected cells was 5000 units above the cutoff level. The meaning of "Down" is *vise verse*. The blank means no significant change.
